# Preferential regulation of duplicated genes by microRNAs in mammals

**DOI:** 10.1186/gb-2008-9-8-r132

**Published:** 2008-08-26

**Authors:** Jingjing Li, Gabriel Musso, Zhaolei Zhang

**Affiliations:** 1 Department of Molecular Genetics, University of Toronto, 1 King's College Circle, Toronto, ON, M5S 1A8, Canada; 2 Donnelly Centre for Cellular and Biomolecular Research, University of Toronto, 160 College Street, Toronto, ON, M5S 3E1, Canada; 3 Banting and Best Department of Medical Research, University of Toronto, 160 College Street, Toronto, ON, M5S 3E1, Canada

## Abstract

**Background:**

Although recent advances have been made in identifying and analyzing instances of microRNA-mediated gene regulation, it remains unclear by what mechanisms attenuation of transcript expression through microRNAs becomes an integral part of post-transcriptional modification, and it is even less clear to what extent this process occurs for mammalian gene duplicates (paralogs). Specifically, while mammalian paralogs are known to overcome their initial complete functional redundancy through variation in regulation and expression, the potential involvement of microRNAs in this process has not been investigated.

**Results:**

We comprehensively investigated the impact of microRNA-mediated post-transcriptional regulation on duplicated genes in human and mouse. Using predicted targets derived from several analysis methods, we report the following observations: microRNA targets are significantly enriched for duplicate genes, implying their roles in the differential regulation of paralogs; on average, duplicate microRNA target genes have longer 3' untranslated regions than singleton targets, and are regulated by more microRNA species, suggesting a more sophisticated mode of regulation; ancient duplicates were more likely to be regulated by microRNAs and, on average, have greater expression divergence than recent duplicates; and ancient duplicate genes share fewer ancestral microRNA regulators, and recent duplicate genes share more common regulating microRNAs.

**Conclusion:**

Collectively, these results demonstrate that microRNAs comprise an important element in evolving the regulatory patterns of mammalian paralogs. We further present an evolutionary model in which microRNAs not only adjust imbalanced dosage effects created by gene duplication, but also help maintain long-term buffering of the phenotypic consequences of gene deletion or ablation.

## Abstract 2

Analysis of duplicate genes and predicted microRNA targets in human and mouse shows that microRNAs are important in how the regulatory patterns of mammalian paralogs have evolved.

## Background

Gene duplication plays an indispensable role in the establishment of genetic novelty, providing not only new genes, and thus the potential for alternative gene functionality, but also facilitating genomic robustness by affording buffering of the consequences of gene deletion [1-3]. While it has been proposed that paralogs often share functions so as to achieve buffering against mutations or deletions [1,2], total redundancy among duplicates is both genetically unfavorable and potentially disruptive to biochemical pathways due to dosage sensitivity [4-6]; thus, a clear understanding of the patterns of divergence between duplicates is crucial in elucidating the mechanisms by which new functions arise.

Previous examinations of gene duplications have assayed their diverging function through comparisons of co-conservation of coding regions [7,8], shared transcriptional regulation [9,10], or similarity in protein-protein [11,12] or genetic [13] interaction partners. One consequent finding is that while paralogs may retain similar functionality, gene expression rapidly diverges immediately after duplication events [10,14,15], suggesting that alterations in gene expression precede potential changes in function. This noted expression divergence currently can not be explained through analysis of transcriptional regulatory motifs [9,10], suggesting that differential regulation at the post-transcriptional level - for example, regulation mediated by microRNAs - could contribute to expression and ultimately functional divergence between gene duplicates. Also, while still speculative, one potential selective benefit of maintaining divergence in expression between functionally overlapping gene duplicates is the possibility for so-called 'expression-reprogramming', or compensation by one paralog upon either deletion or mutation of its sister gene, or in response to specific environmental cues [16]. The mechanism of reported instances of such reprogramming remains unclear, and the potential involvement of post-transcriptional regulation of expression through microRNAs is largely unexplored.

MicroRNAs represent a class of small (typically 22 nucleotides in length) non-coding RNAs that can block translation of their target genes through mRNA degradation or translational repression [17,18]. MicroRNA-mediated regulation at the post-transcriptional level is pervasive in animals, as at least one-third of human genes are estimated to be microRNA targets [19,20]. In animals, microRNA target sites, many of which are highly conserved [21], are generally located in the 3' untranslated region (UTR) of the target mRNA. Despite their obvious importance, little is known about the acquisition of microRNA-mediated regulation.

Considering the pervasive nature of microRNA regulation in mammalian cells, it is intriguing to inquire how the function and evolution of duplicate genes have been modulated by microRNAs. Until recently this area had largely remained unexplored except in the case of plants [22], which have a different microRNA-mediated regulatory system and also are generally more tolerant to polyploidy than animals. Here we describe investigation of the impact of microRNA-mediated regulation on the gene duplication and subsequent functional dispersal of genes in human and mouse. We found that microRNAs are ultimately actively involved in this process, as evidenced by the following: human microRNA targets are significantly enriched for duplicate genes; paralog pairs targeted by microRNAs generally have higher sequence and expression divergence; and duplicated microRNA targets are subjected to a more sophisticated mode of regulation. Furthermore, comparisons of duplicate genes of varying ages suggest that ancient duplicates share few ancestral microRNA regulators. Taken together, our results suggest that microRNA-mediated regulation plays an important role in the regulatory circuits involving duplicate genes, including adjusting imbalanced dosage effects of gene duplicates, and possibly creating a mechanism for genetic buffering.

## Results

### Mammalian microRNA targets are significantly enriched for duplicated genes

In order to determine whether duplicate genes were more likely than singleton genes to be regulated by microRNAs, we first analyzed the genetic composition of known microRNA targets. Specifically, we retrieved all human paralogous gene pairs from Ensembl via BioMart [23], retaining a list of 12,605 genes, each of which has at least one paralog, and 9, 884 singletons genes with no discernable duplicate copy (see Materials and methods). Predicted human microRNA target genes were obtained from the miRGen website [24,25], which contains benchmarked microRNA targets derived from leading prediction programs such as TargetScanS [20] and PicTar[19]. These prediction programs predict microRNA targets based on sequence complementarity, sequence context information, evolutionary conservation, and binding energy (see Materials and methods), and are regarded by previous surveys of microRNA targets as having high confidence [26-29]. However, to further increase the stringency of identified microRNA targets, all analyses presented below have been confirmed using both target sets independently, and only those sites detected jointly by both TargetScanS and PicTar. Additionally, to remove any potential bias caused by the reliance of TargetScanS and PicTar on evolutionary conservation (four-way or five-way conservation used across human, mouse, rat, dog or chicken), we included a third set of predicted human microRNA targets derived from PITA [30], which instead considers only sequence complementarity and site accessibility (see Materials and methods). The PITA prediction program also has the advantage of detecting lineage-specific microRNA targets.

**Figure 1:**
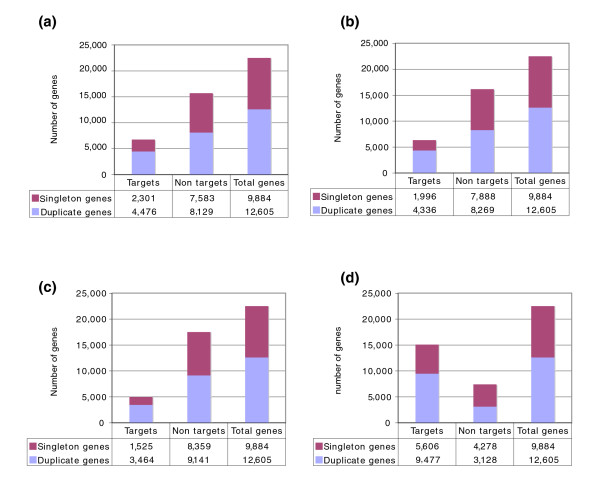
MicroRNA targets are enriched for duplicate genes. This figure shows the number of singleton and duplicate genes among the microRNA targets predicted **(a) **by PicTar, **(b) **by TargetScanS, **(c) **by both programs (intersections), and **(d) **by PITA.

Using each of these stringent datasets, we observed that microRNA targets were significantly enriched for duplicated genes. As shown in Figure 1, duplicate genes are roughly twice as likely to be microRNA targets as comparable singletons (Hyper-geometric test, *p*-values < 5 × 10^-89 ^for the four datasets). As shown in Figure 1a, duplicate genes comprise 56% of all the genes in the genome (12,605 out of 22,489), but make up 66% of the microRNA targets as predicted by PicTar (similar results were found using other target detection methods).

### Enrichment of duplicate genes is not a by-product of longer 3'UTRs, preferential sequence conservation, or gene family expansion

We next sought to determine whether the observed enrichment of duplicated genes for microRNA regulation was in fact an intrinsic property of paralogs, or rather if it was a by-product of another ancillary feature of the analyzed gene set. Specifically, we identified and controlled for three potential sources of biases. First, duplicated genes tended to have longer 3'UTRs than singleton genes (median 878 nucleotides for duplicate genes, versus 850 for singletons, *p *= 4.79 × 10^-4^, two-sided Wilcoxon rank sum test), implying that our observation could have been affected by the greater random chance of duplicates to be detected as microRNA targets. To test this we sampled 5,068 duplicate genes whose 3'UTR length falls into the 0.25 to 0.75 quintiles of the 3'UTR lengths among the 7,595 singleton genes that have available 3'UTR annotation in Ensembl (see Materials and methods), eliminating any biases in the 3'UTR lengths, and repeated our analysis. Again, duplicates were significantly enriched as microRNA targets (*p *< 5.04 × 10^-21^, Hyper-geometric test), eliminating 3'UTR length as a potential source of bias.

The next identified difference between duplicates and singletons stems from the previously reported observation that the sequences of duplicated genes are under more stringent selective constraints [31]. Since many microRNA target sites are known to be evolutionarily conserved [21], it is possible that such an elevated level of overall sequence conservation would have led to an over-representation of duplicate genes among microRNA targets. As microRNA binding sites are predominantly located in 3'UTRs of target genes, we tested the level of sequence conservation downstream of the stop codon in duplicate and singleton genes to determine whether such a bias indeed existed. We compiled 6,937 human duplicate and 4,690 singleton genes, which have available 3'UTR annotations, as well as their mouse orthologs. To determine the divergence between 3'UTR sequences, we adopted the method described in [32,33], whereby we aligned human-mouse orthologous 3'UTR sequences and calculated substitution rates per site (termed as K^3u^) based on the Kimura's two-parameter model. We did not find significant difference in K^3u ^between duplicate genes and singleton genes (median for duplicate genes is 0.191, and median for singleton genes is 0.189; *p *= 0.82, two-sided Wilcoxon rank sum test), indicating that there is no preferential conservation of downstream regions for duplicated genes that could inherently bias our results.

To further confirm that the 3'UTRs of the duplicated and singleton genes are under the same level of selective pressure, we normalized rates of substitution for the 3'UTR of each gene by the corresponding rate of substitution of the coding region in the same gene. Since the number of synonymous substitutions per synonymous site (Ks) in coding sequences is presumably neutral [34], the ratio of K^3u^/Ks can be used to estimate the functional constraints on the 3'UTRs relative to the coding region for individual genes [33]. Upon comparing the K^3u^/Ks ratios between duplicate and singleton genes, we did not observe any statistically significant differences (median of the ratio is 0.311 for duplicate genes and 0.307 for singleton genes; *p *= 0.94, two-sided Wilcoxon rank sum test), suggesting that our observations are not likely influenced by preferential sequence conservation of duplicated genes in 3'UTRs. We also tested our observation on predicted microRNA targets derived from PITA [30], which does not assume evolutionary conservation for the targets. The same enrichment was also observed in this predicted gene set.

Finally, we tested whether the observed enrichment of gene duplicates was evenly distributed among all surveyed paralogs, or rather whether it was influenced by the dominance of a small number of gene families. We clustered the duplicated genes into 3,433 disjoint gene families using single-linkage clustering, then randomly selected one representative gene from each family and compared this set of 3,433 genes with the 9,884 singleton human genes in our dataset. Using each dataset of human microRNA targets, we observed the same enrichment of microRNA targets for duplicate genes (Hyper-geometric test, *p *< 3 × 10^-62^). Collectively, these results demonstrate the robustness of the finding that microRNAs preferentially regulate duplicate genes in human. Similar tests revealed identical findings in mouse, but not *Caenorhabditis elegans *(Additional data file 1), suggesting that it may be a property specific to mammals.

### Duplicated genes exhibit more sophisticated regulatory patterns

**Figure 2:**
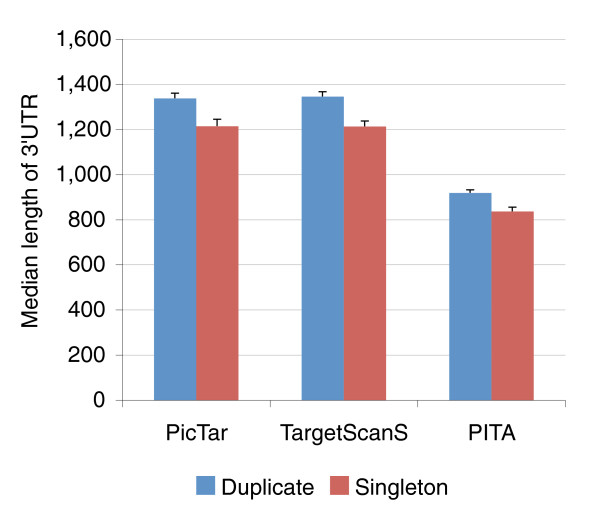
Duplicate genes on average have longer 3'UTR than singleton genes among predicted microRNA targets. Target genes predicted by three computer programs (PicTar, TargetScanS, and PITA) are shown. Error bars indicate standard errors.

Having demonstrated that duplicated genes are more likely to be regulated by microRNAs, we next asked whether any differences existed in the magnitude of microRNA regulation between duplicated and singleton genes. Below we present our analysis based on microRNA targets derived from PicTar; however, all conclusions also hold true for targets derived from TargetScanS, from the intersection between PicTar and TargetScanS, and from PITA. Indeed, we observed that duplicated genes, on average, were regulated by more distinct microRNA species than singletons, as duplicated genes had a median of six distinct microRNA species, while singleton genes had four (*p *< 5 × 10^-14^, two sided Wilcoxon rank sum test). Again, such a disparity could potentially be attributed to differences in the length of 3'UTR between duplicate and singleton genes (Figure 2), as among microRNA target genes predicted by PicTar, TargetScanS or PITA, duplicate genes generally have longer 3'UTRs than singleton genes (*p *< 1 × 10^-5 ^for PicTar targets). However, upon examining the density of target sites in the 3'UTR, defined as the number of distinct microRNA target binding sites types per kilobase, we again observed that duplicated genes had a higher density of microRNA binding sites than comparable singletons (*p *= 6.30 × 10^-5^, two sided Wilcoxon rank sum test), suggesting that paralogs are more actively regulated by microRNAs than singletons.

### Divergence in miRNA regulation between paralogs

**Figure 3:**
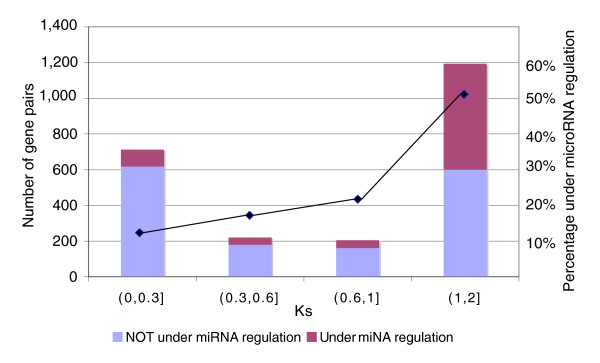
The distribution of duplicate gene pairs among four Ks intervals. Duplicate pairs were grouped according to their pair-wise Ks divergence from the smallest to the greatest. It is clear that ancient duplicates with higher Ks values have a higher chance to be regulated by microRNAs.

We next divided the paralogs into two groups: those pairs without microRNA regulation (that is, neither of the two genes is a microRNA target; 1,561 pairs); and pairs with at least one copy regulated by microRNAs (771 pairs). Ks values were then tallied between paralogs as a proxy for age since duplication (values used were between 0.05 and 2 as Ks beyond this range implies either too little or saturated sequence divergence, making the resulting inferences unreliable). To investigate any correlations between age of the duplicates and microRNA mediated regulation, both microRNA-regulated and non-regulated paralog pairs were sub-divided into four categories based on their pairwise Ks values (Figure 3). We found that pairs with greater Ks, and thus those with greater time since duplication, were more likely to be regulated by microRNAs (mean Ks = 0.78 for the pairs without miRNA regulation, compared with mean Ks = 1.38 for pairs with miRNA regulation; *p *= 4.8 × 10^-84^, two-sided Wilcoxon rank sum test), suggesting that duplicated genes can acquire microRNA regulation over time.

**Figure 4:**
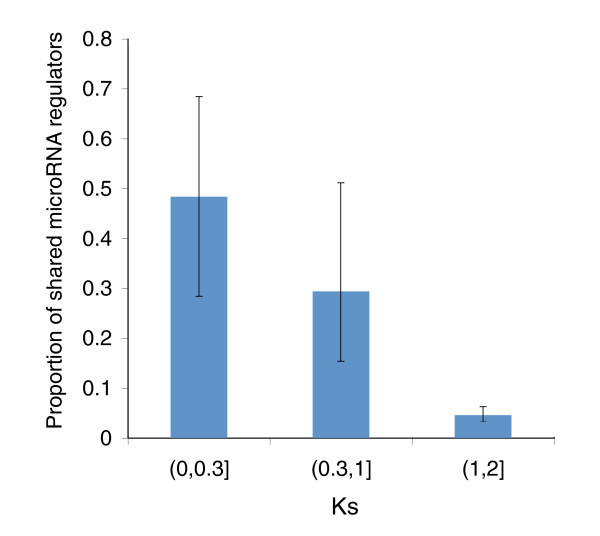
Duplicate gene pairs with greater Ks usually share few microRNA regulators. The mean overlap scores and 95% confidence intervals are shown for each Ks interval. The 95% confidence intervals were derived from 5,000 bootstrap re-sampling.

Next we investigated the extent to which the paralog pairs share common microRNA regulators, presumably those inherited from their ancestral parental genes. For duplicate gene pairs in which both paralogs were regulated by at least one microRNA (224 in total) we defined the overlap score of shared microRNA regulators as the ratio between the number of common microRNA regulators (intersection) and all the total regulators for the pair (union). We observed a significant negative correlation between this overlap score and the Ks values for paralog pairs (r = -0.56, *p *= 3.4054 × 10^-20^, Pearson correlation), intuitively implying that more recent paralogs share proportionally more common microRNA binding sites (Figure 4), and consequently that ancestral microRNA regulation patterns are lost by ancient duplicates.

Finally, to determine if differences in microRNA regulation in fact correlated with varied expression, we obtained human gene expression data across 79 tissues [35], and compared these versus annotated target data. After mapping probe set names to Ensembl gene IDs (1,388 pairs had expression data mapped to Ensembl IDs, 575 of which where at least one paralog was a microRNA target; see Materials and methods), expression divergence was calculated for each pair as 1 minus the Pearson correlation of expression across all tissue types. Consistent with what was observed regarding sequence divergence, duplicate gene pairs regulated by microRNAs had more divergent expression profiles (mean expression divergence is 0.82, compared with 0.57, *p *= 5.62 × 10^-20 ^for duplicate pairs without microRNA regulation, two-sided Wilcoxon rank sum test), suggesting that differences in microRNA-mediated regulation are likely ultimately manifested as altered gene expression between paralogs.

## Discussion

In ancient duplicate gene pairs regulated by microRNAs, sister paralogs seemingly have largely evolved varying sets of microRNA regulators, either through acquisition of novel binding sites or through the loss of ancestral ones. As we observed that microRNA targets with duplicate copies were generally under more sophisticated regulation mediated by microRNAs, we postulate that microRNA regulation is selectively advantageous among higher organisms, and might provide the groundwork for additional regulatory and buffering mechanisms. This can be potentially explained when considering the selective pressures incident on duplicated genes following duplication.

Relaxed selective pressure acting on duplicates, especially on the 3'UTRs, and the subsequent accelerated evolution may have ultimately led to the emergence of additional microRNA binding sites. As noted previously [7,36], immediately following a duplication event paralogs experience accelerated evolution in sequence, function and regulation due to relaxed selective constraints. It is conceivable that the 3'UTR region of the duplicate genes could have evolved at a faster rate than comparable singleton genes, allowing expedition of microRNA target site gain [26]. Additionally, such enrichment could be potentially beneficial to the organism since it offers an additional mechanism to regulate protein production from duplicate genes, thus avoiding complications of dosage imbalance, which may potentially be detrimental to the organism [4-6], adding additional pressure for 3'UTR modification.

Another selective advantage of adapting microRNA-mediated regulation is the potential involvement in compensatory buffering mechanisms among duplicates [1-3]. Kafri and colleagues previously used the mouse paralogs *Myod1*(alias: *MyoD*) and *Myf5 *(alias: *Myf-5*) as an example to illustrate genetic buffering (see the supplemental materials in [16]), both of which are regulated by a number of microRNAs. These genes are transcription factors that show divergent expression patterns [16,37,38], yet deletion of *Myod1 *leads to the up-regulation of its sister paralog *Myf5*. Below we present a model whereby similar paralogs may have adapted compensatory buffering mechanisms through mediating microRNA regulation.

### A model for microRNA mediated genetic buffering

**Figure 5:**
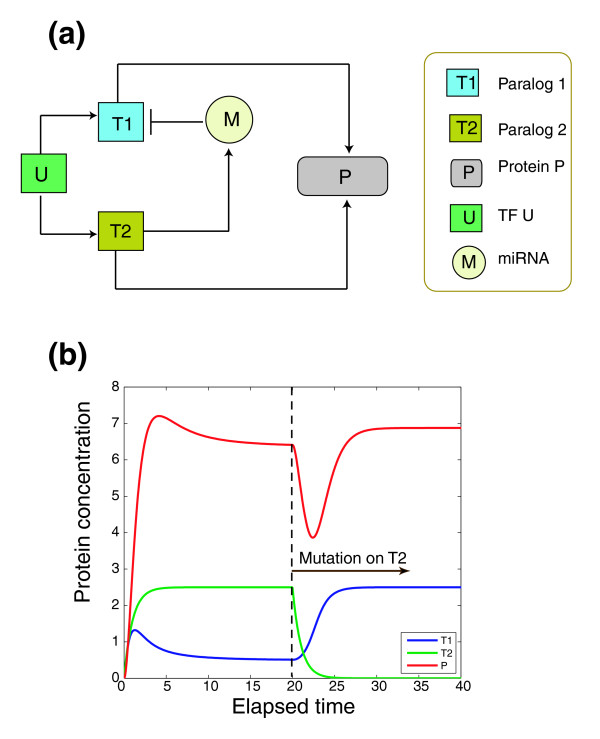
Dynamic simulation of regulation of duplucate genes by microRNA. **(a) **A schematic diagram of a hypothetical microRNA-mediated regulatory circuit involving duplicate genes. A detailed explanation of the elements is in the main text. **(b) **Simulation of a microRNA-mediated regulatory circuit as depicted in (a). Prior to the null mutation on *T2 *at time point 20, the level of *T1 *is repressed by microRNA *M*, and the expression of *P *is mainly regulated by *T2*. After the null mutation on *T2*, *T1 *is up-regulated, which in turn restores *P *up to its original level.

In the following we extend the reprogramming hypothesis originally derived from yeast to mammals by proposing a kinetic model in which the microRNA-mediated post-transcriptional regulation can facilitate the genetic buffering between gene duplicates. As shown in Figure 5a, under the reprogramming hypothesis, paralog genes *T1 *and *T2 *are regulated by a common set of transcription factor(s), denoted as *U*. Protein products of both *T1 *and *T2 *regulate the target protein *P*. However, the effect of *T1 *regulation on *P *is attenuated by a microRNA (*M*), which is either hosted or activated by *T2*. If *T2 *is down-regulated due to mutations or deletions, the expression of microRNA *M *will be repressed, which elevates the expression of *T1 *so as to maintain a similar expression level of *P*. Mathematically, the topology of our proposed kinetic model is intrinsically stable with only one steady state that corresponds to a dynamic equilibrium in protein concentration (Figure 5b).

According to the simulated dynamics, initially, due to the effect of *M*, the protein level of *T1 *(blue line) is repressed and the highly expressed *T2 *promotes the protein level of *P*. Upon the null mutation of *T2*, the protein concentration of *T1 *is up-regulated until reaching a steady state. Meanwhile, with up-regulation of *T1*, after a transient down-regulation of *P *caused by the mutation of *T2*, the level of *M *is promptly restored to its original level.

A number of examples within the literature exemplify this model. For example, *Oct4 *(HGNC symbol: *Pou5f1*, POU class 5 homeobox 1) is one of the three genes comprising the core regulatory circuitry in human embryonic stem cells [39]. Previous experiments have shown that *Oct4 *can affect transcription of the microRNA mir-301 [39], which in turn targets the *Oct4 *paralogs *Pou4f1*, *Pou3f2 *and *Pou4f2*. Despite the crucial role of *Oct4 *in human embryonic stem cells [40], recent research has shown that no phenotypic changes could be observed upon the mutation of *Oct4 *as "*Oct4 *is dispensable for both self-renewal and maintenance of somatic stem cells in the adult mammal" [40]. It is possible, however, that living cells have evolved a buffering mechanism whereby the loss of *Oct4 *down-regulates *mir-301*, which in turn up-regulates its paralogs *Pou4f1*, *Pou3f2 *or *Pou4f2*, which compensate for the function of *Oct4*.

### MicroRNAs, genome duplications, and morphological complexity

There is an increasing amount of evidence that whole genome duplication events actually occurred twice during the emergence of vertebrates [41-43], being a major source of morphological complexity among vertebrates. However, a recent survey of the distribution of microRNA families among a wide range of chordate species by Heimberg and colleagues [44] cast doubt on the presumed importance of such duplication events. In this study, a dramatic expansion of microRNA families was observed at the base of the vertebrates (prior to the divergence between lamprey and jawed fishes but after the divergence between vertebrates and other chordates, and thus prior to when the ancient whole genome duplication events are thought to have occurred), which purportedly had a greater role than genome duplication in creating the extensive morphology complexity among extant vertebrates. Regardless of what triggered the expansion of microRNA families, we argue that both the microRNA expansion and the genome duplication events, and perhaps most likely the synergistic combination of the two, were responsible for generating the enrichment of duplicate genes among microRNA targets observed here.

While a microRNA expansion event created an abundance of regulators to evolve into an elaborate regulatory network, subsequent genome duplication(s) may also have provided additional genes as effectors for the newly generated microRNAs to operate on. Furthermore, the potential relaxed selective pressure following genome duplication events would have further facilitated genes gaining microRNA binding sites. This reasoning is supported by our observation that microRNA targeting bias towards duplicate genes might be unique in high order organisms (human and mouse) but not in lower organisms such as *C. elegans*.

Takuno and Inna [22] recently surveyed the affect of microRNAs on the expansion and evolution of gene families of *Arabidopsis thaliana*, which has undergone multiple whole genome duplication events. These authors reported that gene families consisting of multiple paralogous genes tended to be regulated by fewer microRNAs in *Arabidopsis*, which is seemingly different from what we observed in human as our results suggest paralogous genes are under more sophisticated microRNA regulation. However, we believe such inconsistency can be explained by the fundamental differences in microRNA-mediated regulation between plants and mammals. Animal microRNAs can only bind to target sites that are located in the 3'UTR of genes, whereas plant microRNAs can bind 5'UTR and coding regions as well [17]. In addition, in plants, microRNAs and their target sites usually require perfect base-pairing, whereas one or two mismatches are generally tolerated in animals, resulting in far more prevalent microRNA regulation in animals (human microRNAs are predicted to regulate hundreds of genes while microRNAs in plants generally regulate much fewer target genes). In addition, plants presumably are more tolerant of gene duplications as plants frequently undergo whole-genome duplication and polyploidization events [45]. Together, these differences suggest different mechanisms for both microRNA family expansion and adaptation of gene regulatory mechanisms in plants.

## Conclusion

It is widely acknowledged that expression divergence increases proportionally with the increase of divergence time between sister paralogs [15,46]. However, studies of the mechanism of divergence have focused mainly on transcription factor mediated regulation [8,9,11]. Here, we demonstrate that human microRNA target genes are significantly enriched for duplicate genes, and also that duplicate pairs with greater divergence, while having a higher chance to be regulated by microRNAs, share very few common microRNA regulators. This difference in microRNA regulation likely plays a role in the observed expression difference between these same duplicates. By eliminating potential confounding factors, our observations strongly suggest that the microRNA could potentially affect functional divergence between paralog pairs, and high-order organisms have adopted microRNA as an efficient and sophisticated mechanism to control and modulate protein production from duplicate genes.

MicroRNA regulation is not considered to be a simplistic process, and likely requires more detailed evolutionary and functional models before a full understanding can be gleamed. For example, additional factors, such as mRNA splicing, polyadenylation, and chromatin modifications, offer new paths to further investigate the impact of combinatorial regulation at multiple levels. Regulation by microRNA can also be adapted in response to common cellular processes, as recently evidenced in cell-cycle arrest [47], indicating possible responses to dynamic influences. Currently, while high-quality fully assembled genome sequences are available for a number of vertebrates, comprehensive and accurate annotation of protein-coding genes and microRNAs has been done only for human and mouse. Once the quality and quantity of genome sequences and annotations are improved, it will be possible to test whether the same enrichment and other patterns can also be observed in other vertebrates. Yet, the simple model provided here provides sufficient groundwork to begin testing, and ultimately understanding, the evident impact that microRNAs play in gene duplication and subsequent functional differentiation.

## Materials and methods

### Compilation of human genes

The complete set of human duplicate genes was compiled from Ensembl database (version 46) through seven steps, including best reciprocal Blast search, sequence clustering, multiple alignment and phylogenetic analysis. A description of the procedures can be found at the Ensembl database web site. We also downloaded all human genes from Ensembl via the BioMart utility. After removing redundant paralog pairs (that is, in the case of A-B and B-A, we retained only A-B) and selecting only those pairs for which both genes are annotated as 'known genes', we retained a final total of 39,177 paralog pairs. This corresponds to a total of 12,605 unique genes that have at least one duplicate copy in the human genome (note that one gene can have multiple paralogs. In addition, we also retained a total of 9, 884 known singleton genes that have no duplicate copy.

### The compilation of microRNA target genes

We retrieved the genome-wide computationally predicted human microRNA targets at the miRGen website [24,25], which pre-compiled and benchmarked the up-to-date target predictions from leading algorithms, including TargetScanS and PicTar, which we used in this study. The accuracy of these prediction sets had been previously benchmarked by gene expression profiling with high confidence [48], and have been used in a number of recent publications [26-29]. The input human genes used in these prediction programs are largely consistent with the most current Ensembl annotations, as only less than 30 genes did not have concurrent IDs in Ensembl version 46. After removing those defunct Ensembl IDs, the total number of predicted targets was 6,777 for PicTar, 6,332 for TargetScanS, and 4,989 for their intersection. In addition to using PicTar and TargetScanS, we also confirmed our conclusion presented here based on a set of newly released microRNA targets derived from PITA [30]. We downloaded human microRNA targets from the PITA Targets Catalog (no flank) from the Weizman Institute website [49], mapped the gene symbols to Ensembl IDs and retained a final list of 15, 083 genes annotated as microRNA targets in PITA. Note that unlike predictions from PicTar and TargetScanS, PITA made predictions based on sequence features and site accessibility instead of using cross-species conservation; thus, many non-conserved microRNA targets are included in the list, so many more genes are annotated as microRNA targets, especially as targets for primate-specific microRNAs. All the miRNA targets used in this study are listed in Additional data file 2.

### Sequence conservation in 3'UTRs

A list of human-mouse one-to-one orthologs were downloaded from Ensembl version 46, which included 8,581 human genes with at least one duplicate copy and 5,777 singleton genes having no duplicate copy. We also downloaded 3'UTR sequences and coding sequences of all the known genes for human and mouse in Ensembl version 46. For genes that are annotated as having multiple 3'UTR sequences, we retained the longest one. Thus, our final list included 6,937 duplicate genes and 4, 690 singleton genes with available 3'UTR sequences for them and their mouse orthologs. To determine sequence conservation in 3'UTRs, we adopted methods described in [32,33]. Briefly, we first aligned human-mouse orthologous 3'UTR sequences and then calculated the substitution rates per site (K^3u^) based on the Kimura two-parameter model [50]. Similarly, we also aligned human-mouse orthologous coding sequences, and implemented YN00 in the PAML package [51] to calculate the number of synonymous substitutions per synonymous site (Ks).

### Extraction of independent duplicate pairs

For all the non-redundant 12,605 duplicate genes defined in Ensembl (see above), we retrieved their coding sequences and used ClustalW [52] to realign the 39,177 paralog pairs. With the pair-wise alignment, we also implemented YN00 in PAML [51] to calculate the rates of synonymous (Ks) and non-synonymous (Ka) substitutions per site and sequence identity of the aligned regions. Of the 39,177 gene pairs, we excluded 10 pairs that have a stop codon within the coding region. Similar to previously described procedures [36,53], we clustered the 39,177 pairs into 3,433 gene families, and in each gene family, we selected duplicate pairs from the lowest Ks to the highest Ks values based on two criteria: once a pair is selected, the genes of the pair cannot be selected again; and the selected pairs should have a Ks between 0.05 and 2. Our final list consisted of 2, 332 independent duplicate pairs satisfying these criteria (Additional data file 3).

### Human gene expression data

We retrieved the Novartis human gene expression data across 79 tissue types from the web [54]; the data from U133A+GNF1H (gcRMA) chips were used in this study. Using the annotation file for the GNF1H chip and the name-mapping table for U133A chip from Ensembl version 46, we mapped the probe names used in the microarray experiments to Ensembl identifiers; the expression intensities of multiple probes that correspond to one gene were averaged. When calculating expression correlation, the dataset was normalized as Z-scores (median-centered with one standard deviation) for each tissue (cell-type).

### Dynamic simulation

We attempted to computationally simulate the genetic reprogramming model as defined in Figure 5a[16]: *U *denotes a common regulator that activates the transcription of *T1 *and *T2*; *T1 *and *T2 *encode proteins that in turn regulate protein *P*, either transcriptionally or translationally. *T2 *is also assumed to activate the expression of microRNA *M*, which in turn down-regulates *T1*. We used the following two sets of equations to describe the dynamics of the regulatory circuits before and after a null mutation on *T2*.

dT1dt=k1U−αT1−kmMT1dT1dt=k1U−αT1−kmMT1dT2dt=k2U−βT2dT2dt=−βT2dMdt=−kmMT+1ktT2dMdt=−kmMT+1ktT2dPdt=kp1T1+kp2T2−γPdPdt=kp1T1+kp2T2−γP

The two dynamic systems as we proposed here are intrinsically stable and each has a steady state. *T*_1_, *T*_2_, *M *and *P *are concentrations as indicated in Figure 5a. Based on the assumption of a quasi-steady state, the degradation rates were set as *α *= *β *= *γ *= 1. The reaction rates were set as: *k*_1 _= *k*_2 _= 0.2, *k*_*t *_= 0.8, *k*_*p*1 _= 2.75 and *k*_*m *_= *k*_*p*2 _= 2. We also set *U *= 12.5 and the concentration of other molecules are 0. The mutation on *T2 *was set at the 20 time point, so the steady state of the first dynamic system (before *T2 *mutation) is the initial condition for the second dynamic system (after *T2 *mutation).

## Abbreviations

UTR: untranslated region.

## Authors' contributions

JL and ZZ designed the study. JL collected data, carried out the calculations, and performed statistical analyses. GM participated in the analysis and revised the manuscript. JL and ZZ wrote the manuscript. All authors read and approved the final manuscript.

## Additional data files

The following additional data are available with the online version of this paper. Additional data file 1 is a set of figures describing the analysis of the duplicate genes in mouse and *C. elegans*. Additional data file 2 is an Excel spreadsheet listing the predicted human microRNA target genes used in this work. Additional data file 3 is an Excel spreadsheet listing the independent duplicate gene pairs and their Ks values.

## Supplementary Material

Additional data file 1 Detailed analysis of duplicated genes in mouse and *C. elegans*. Click here for file

Additional data file 2 The list of human microRNA targets used in the work. Click here for file

Additional data file 3 The independent pairs with Ks values and group information. Click here for file
