# Supplementary material for: Preferential regulation of duplicated genes by microRNAs in mammals
Source: Genome Biol. 2008 Aug 26;9(8):R132. doi: 10.1186/gb-2008-9-8-r132 (PMC2575522; doi:10.1186/gb-2008-9-8-r132)
Supplement: Additional data file 1 — Detailed analysis of duplicated genes in mouse and C. elegans. [file gb-2008-9-8-r132-S1.doc]

**The enrichment of duplicate genes in microRNA targets of mouse**

With the homology annotation in Ensembl 46, we compiled 13,601 known mouse genes as duplicate genes and 9,521 genes as singleton genes. As there is no available target prediction in mouse from PicTar compiled in miRGen database: <http://www.diana.pcbi.upenn.edu/miRGen.html>, we first performed our analysis with the microRNA targets derived from TargetScanS. We mapped the gene symbols to Ensembl IDs, and finally retained 4805 genes having Ensembl IDs, in which 68 Ensembl IDs are defunct in the release of Ensembl 46. Thus we used 4,737 mouse microRNA targets derived from TargetScanS with Ensembl IDs. The comparison result is shown in Figure S1. The targeting bias towards duplicate genes is significant with P=1.4×10^-45^


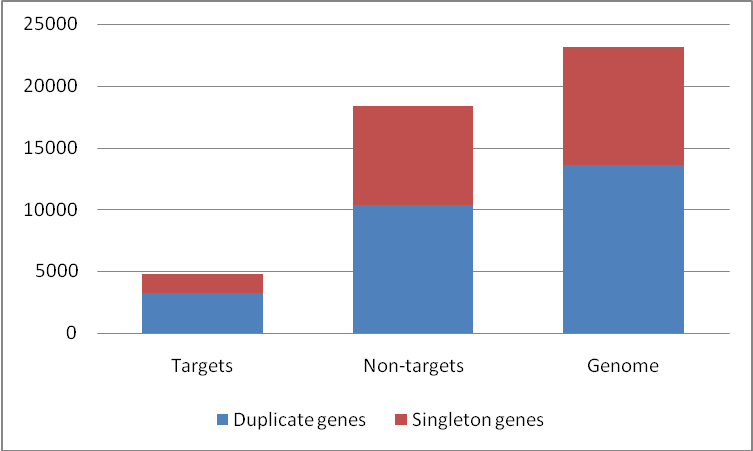


**Figure S1**. Duplicate genes are over-enriched in mouse target genes. The mouse target genes were from TargetScanS database.

Secondly, we confirmed the enrichment of duplicate genes in microRNA targets derived from PITA, which used site accessibility to predict microRNA targets instead of using evolutionary conservation as adopted in PicTar and TargetScanS, again we found the same trend with P=7×10^-36^.

**Duplicate genes in microRNA targets of *C.elegans***

Similarly, we compiled 8,135 duplicate genes and 11,910 singleton genes in *C.elegans* genome from the homology annotations provided by Ensembl, and we only retained those pairs, for which both members are annotated as known genes. As described in the main text, we also downloaded the microRNA targets of *C.elegans* from <http://www.diana.pcbi.upenn.edu/miRGen/>, in which there are pre-compiled microRNA targets derived from PicTar, TargetScanS and their intersections. By removing the defunct gene IDs, we retained a total of 2,692 predicted microRNA targets of *C.elegans*. Contrary to our observation on human and mouse, as shown in Figure S2, in *C.elegans*, we did not find the enrichment of duplicate genes in microRNA targets, with P~=1. This trend was further confirmed by using microRNA targets derived from TargetScanS and PITA.


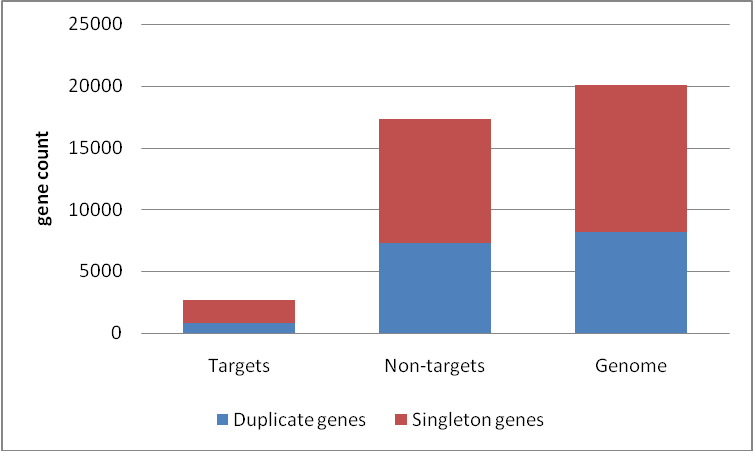


**Figure S2**. Duplicate genes are not enriched in microRNA targets of C.elegans. Targets were derived from PicTar dataset.

Collectively, we observed enrichment of duplicated genes in microRNA targets for human and mouse genomes, but cannot observe the enrichment in lower organisms, such as *C.elegans* which suggests that microRNA regulation for duplicated genes might be unique in higher-order organisms.
